# Transcriptome analysis of ankylosed primary molars with infraocclusion

**DOI:** 10.1038/s41368-019-0070-1

**Published:** 2020-02-21

**Authors:** Annie Tong, Yuh-Lit Chow, Katie Xu, Rita Hardiman, Paul Schneider, Seong-Seng Tan

**Affiliations:** 1 Melbourne Dental School, The University of Melbourne, Melbourne, Australia; 2 Florey Institute of Neuroscience, The University of Melbourne, Melbourne, Australia

**Keywords:** Malocclusion, Malocclusion, Malocclusion, Paediatric dentistry

## Abstract

Primary molar ankylosis with infraocclusion can retard dental arch development and cause dental asymmetry. Despite its widespread prevalence, little is known about its molecular etiology and pathogenesis. To address this, RNA sequencing was used to generate transcriptomes of furcal bone from infraoccluded (*n* = 7) and non-infraoccluded (*n* = 9) primary second molars, all without succeeding biscuspids. Of the 18 529 expressed genes, 432 (2.3%) genes were differentially expressed between the two groups (false discovery rate < 0.05). Hierarchical clustering and principal component analysis showed clear separation in gene expression between infraoccluded and non-infraoccluded samples. Pathway analyses indicated that molar ankylosis is associated with the expression of genes consistent with the cellular inflammatory response and epithelial cell turnover. Independent validation using six expressed genes by immunohistochemical analysis demonstrated that the corresponding proteins are strongly expressed in the developing molar tooth germ, in particular the dental follicle and inner enamel epithelium. The descendants of these structures include the periodontal ligament, cementum, bone and epithelial rests of Malassez; tissues that are central to the ankylotic process. We therefore propose that ankylosis involves an increased inflammatory response associated with disruptions to the developmental remnants of the dental follicle and epithelial rests of Malassez.

## Introduction

Dental ankylosis is defined as a fusion of cementum or dentine with alveolar bone.^1^ Primary molar ankylosis can cause severe clinical consequences in the growing child, including:^1–4^ (1) Tooth infraocclusion and vertical bone defect, (2) Tipping of adjacent teeth into the space of infraocclusion, causing loss of arch space, dental asymmetry, midline deviation,^4^ and impaction of the ankylosed tooth and its successor, (3) Supra-eruption of opposing teeth, and (4) Deflected path of eruption of successors, with displacement in the form of tipping and ectopic eruption of successors.

Despite the abundance of clinical and epidemiological data, little is known about the molecular correlates of primary molar ankylosis. Genetic associations have been proposed, mostly based on epidemiological data from familial, ethnicity, and dental anomaly pattern studies.^5–10^ However, despite compelling evidence for strong familial and ethnic associations, no candidate genes or molecular pathways have been identified.

A number of studies have tested hypotheses of molecular pathways of ankylosis. Prime among them is the dysregulation of proteins involved in hard tissue turnover. For example, descriptive studies of fixed tissues suggest altered expression of osteoprotegerin (OPG), receptor activator of nuclear factor kappa-β (RANK), and RANK ligand (RANKL) in a rat model of ankylosis induced by thermal trauma using dry ice.^11^ PDL space mineralization and dental ankylosis have been observed in animals with altered bone metabolism, for example in mutant mice with elevated Wnt signalling,^12^ in osteopetrotic mutant rabbits with reduced osteoclast-mediated bone resorption,^13^ and in mice injected with bisphosphonate.^14^ A study of five human subjects with loss-of-function mutations in *ENPP1* associated with generalized arterial calcification of infancy (GACI) found increased cementogenesis and reported histories of infraocclusion and ankylosis.^15^ On the other hand, in a staining study, bone from ankylosed human primary molars demonstrated no difference in the expression of NADH-diaphorase, acid phosphatase, and alkaline phosphatase.^16^

The above studies suggest that animal models can provide useful insights into the functions of selected proteins and pathways, but are not representative of the ankylotic process in humans, which is unique in that it is not induced by trauma, is seemingly idiopathic and spontaneous, and has high prevalence (e.g. 22% in Finland^17^ and 38% in Israel^18^) that cannot be explained by rare genetic mutations. So while these candidate gene and protein approaches can be informative, they are hampered by scale and scarcity of established candidates. Thus, it is timely to approach the problem using human transcriptome-wide analysis.

In this study, RNA sequencing (RNA-seq) was used to characterize and compare the transcriptome profiles of primary molars with and without infraocclusion. Following analysis, a small number of differentially expressed (DE) genes were examined for their protein distribution in developing tooth germs.

## Results

### DE genes in bone tissue

**Fig. 1:**
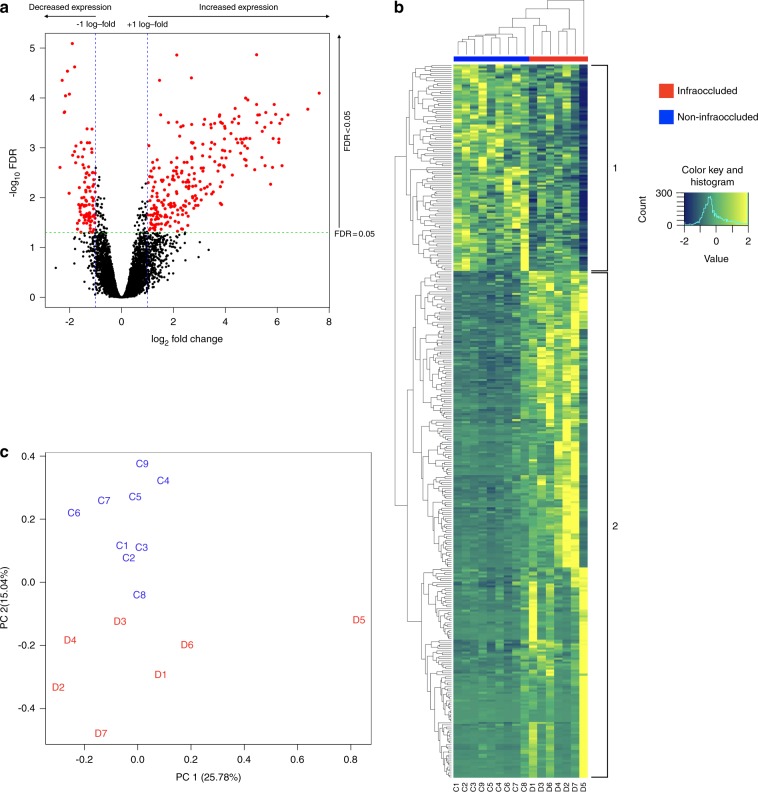
Visualization of gene expression using volcano plot, hierarchical clustering, and principal component analysis. **a** All 18 529 genes were plotted as individual dots on a graph of log_2_ fold change (logFC) versus negative log_10_ FDR. At cut-offs of FDR <0.05 (marked by green dotted line) and logFC of ≤−1 or ≥1 (marked by blue dotted lines), 356 genes were differentially expressed (marked by red dots): 253 genes had increased expression (red dots right of blue dotted line), and 103 genes had decreased expression (red dots left of blue dotted line) in the infraoccluded group compared to the non-infraoccluded group. **b** Hierarchical clustering and heatmap of 432 DE genes (FDR < 0.05). Infraoccluded (D1–D7) and non-infraoccluded (C1–C9) samples clustered into their respective groups. Each row in the heatmap denotes one gene, with the expression level ranging from low (blue) to high (yellow); hence each gene’s consistency of expression across all samples can be visualized. Gene clusters 1 and 2 denote the genes that had decreased and increased expression, respectively, in the infraoccluded group compared to the non-infraoccluded group. **c** Principal component analysis was performed across all 18 529 identified genes. All infraoccluded (D1–D7, coloured red) and non-infraoccluded (C1–C9, coloured blue) samples separated into their respective groups at approximately −0.1 in principal component 2 (PC2). Greater variation in the infraoccluded group compared to the non-infraoccluded group is evidenced by greater scatter of infraoccluded samples on the PCA plot

After excluding genes with low counts (see the “Materials and methods” section), 18 529 genes were retained for further analysis, from which 432 genes (2.3%) were found to be differentially expressed between the two groups (false discovery rate (FDR) < 0.05) (Supplementary Table 1). Of the 432 DE genes, all except two had log_2_ fold change (logFC) of ≤−0.59 or ≥0.59 (i.e. exhibiting 1.5-fold change or more), and 356 genes had logFC of ≤−1 or ≥1 (i.e. twofold change or more) (Fig. 1a). Hierarchical clustering and heatmap analysis clustered all samples into their respective experimental and control groups, indicating clear differences between the two groups (Fig. 1b). Furthermore, there was greater variation between samples in the infraoccluded group compared to the non-infraoccluded group. This was evidenced by infraoccluded samples clustering at higher points on the dendrogram (the higher up the linkage between clusters occurs, the greater the difference between these clusters). The greater variation in the infraoccluded group was also demonstrated by Principal component analysis (PCA). The first two principal components, which accounted for 40.82% of the total variance of the data, separated all samples into their respective two groups (Fig. 1c).

**Fig. 2:**
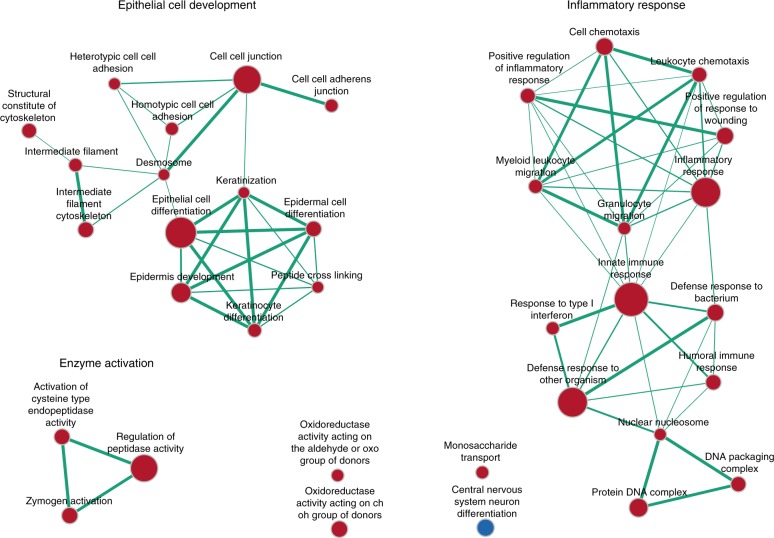
Enrichment map of significant GO pathways created using Cytoscape. Two main pathway clusters were found: (1) Epithelial cell differentiation, keratin expression, and cell junction activity, and (2) Inflammatory response. A third cluster related to non-specific enzyme activation. Each node represents a significantly enriched pathway (FDR < 0.05). Red nodes represent positive enrichment in infraoccluded samples (i.e. increased expression in infraocclusion), while blue nodes represent negative enrichment in infraoccluded samples (i.e. decreased expression in infraocclusion). Size of node represents number of genes enriching the pathway. Edges represent pathway overlap (i.e. genes common to both pathways), and width of edges represents size of overlap (i.e. number of genes common to both pathways) (overlap coefficient > 0.25)

To identify specific biological pathways that may be linked to infraocclusion, Gene set enrichment analysis (GSEA) and Ingenuity Pathway Analysis (IPA) were performed. GSEA produced 36 significantly enriched gene ontology (GO) pathways (FDR < 0.05). Pathway size (i.e. number of genes in dataset enriching each pathway), overlap (i.e. number of genes in dataset common to two pathways), and positive/negative enrichment were separately mapped (Fig. 2). Two principal clusters were found, relating to (1) Epithelial cell differentiation, keratin expression, and cell junction activity, and (2) Inflammatory response.

**Fig. 3:**
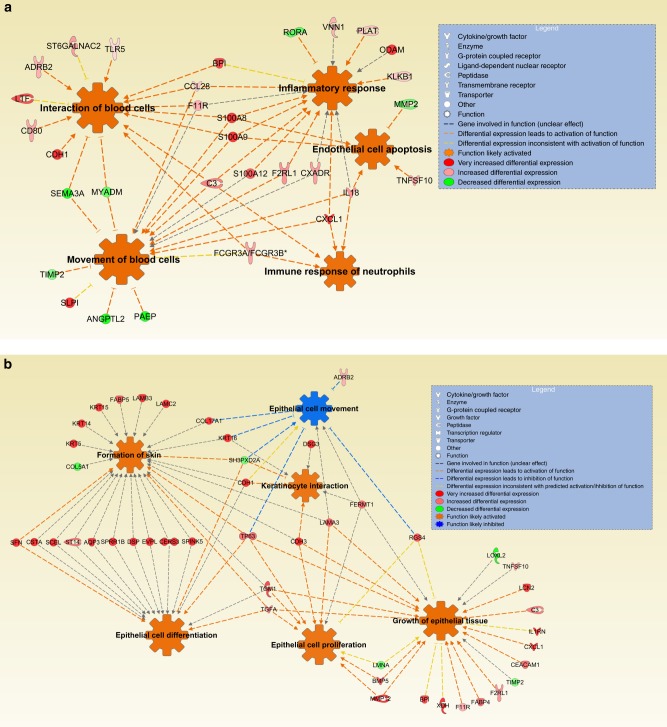
IPA found DE genes in infraocclusion. They were associated with **a** increased activation of inflammatory response and recruitment of leukocytes, and **b** increased activation of epithelial cell proliferation and differentiation, and inhibition of epithelial cell movement. Due to the hierarchical nature of classifying pathways, only the higher-level pathways are illustrated (lower pathways that are subsets of higher pathways are not shown separately)

IPA produced 20 significantly activated or inhibited downstream functions (*z*-score ≥ 1.7 or ≤−1.7) (Supplementary Table 2). The functions could be categorised into two broader functions: (1) Activation of inflammatory response and recruitment of leukocytes (Fig. 3a); and (2) Activation of epithelial cell proliferation and differentiation, and inhibition of epithelial cell movement (Fig. 3b).

### Immunohistochemical detection of proteins encoded by DE genes in tooth germs

Immunohistochemistry was conducted to validate the spatial expression of DE genes in tooth structures. Of the six genes under consideration, three were over-expressed, and three were under-expressed, in infraocclusion. These genes were selected based on the criterion that antibodies to their proteins were commercially available, not because they exhibited the greatest fold-change or any other variable.

**Fig. 4:**
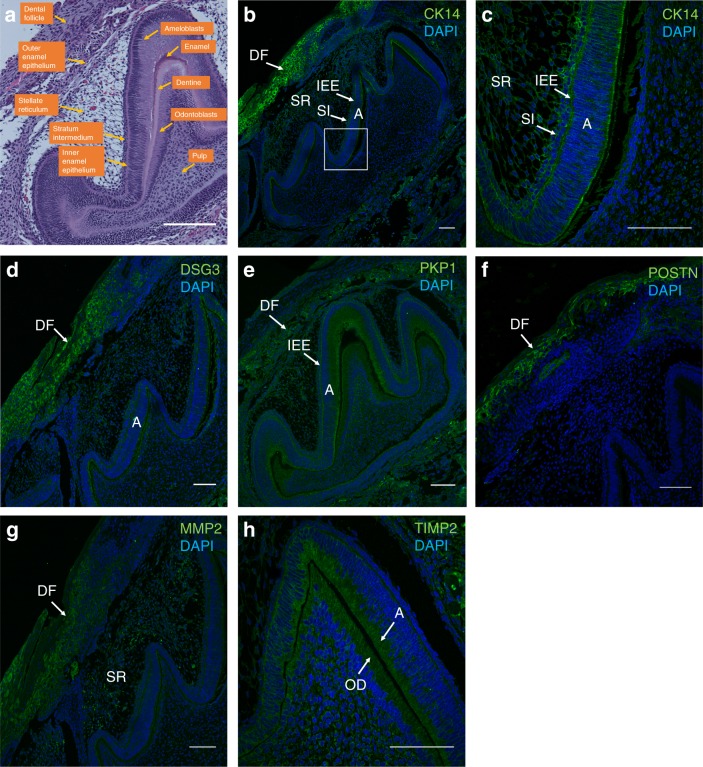
Immunohistochemical staining of proteins encoded by over-expressed (CK14, DSG3, PKP1) and under-expressed (POSTN, TIMP2, MMP2) genes in infraocclusion. **a** Hematoxylin and Eosin staining of P1 mouse molar tooth germ in the sagittal plane with various nascent structures within the dental follicle. **b** CK14 was strongly stained in the dental follicle. **c** Higher magnification of boxed area showed CK14 immunostaining in the stellate reticulum, stratum intermedium, inner enamel epithelium and ameloblast layer. **d** DSG3 was strongly stained in the dental follicle and to a lesser extent in the ameloblast layer. **e** PKP1 was strongly stained in the dental follicle, as well as the inner enamel epithelium and the apical aspect of the ameloblast layer. **f** POSTN was immunostained only in the dental follicle. **g** MMP2 was expressed only in the dental follicle and stellate reticulum. **h** TIMP2 was the only protein in this sample not expressed in the dental follicle, but was abundantly present in the ameloblast and odontoblast layers. Scale bars are 100 μm

The results (summarized in Table 1) showed that the dental follicle was the most common site of protein expression, with five out of the six selected proteins involved. Three proteins (CK14, DSG3, TIMP2) were from the infraoccluded group and two proteins (POSTN and MMP2) from the control group. CK14 (over-expressed in infraocclusion) was strongly stained in the dental follicle (Fig. 4b) as well as in the inner enamel epithelium, stratum intermedium, stellate reticulum and ameloblast layer (Fig. 4c). DSG3 (over-expressed in infraocclusion) was also strongly stained in the dental follicle as well as in the ameloblasts layer (Fig. 4d). PKP1 (over-expresssed in infraocclusion), while strongly expressed in the dental follicle, was also expressed in the inner enamel epithelium and ameloblasts (Fig. 4e). In contrast, POSTN (under-expressed in infraocclusion) was stained solely in the dental follicle and not elsewhere in the dental organ (Fig. 4f). MMP2 (under-expressed in infraocclusion) was similarly active predominantly in the dental follicle but also in the stellate reticulum (Fig. 4g). Of the six proteins, only TIMP2 (under-expressed in infraocclusion) failed to be found in the dental follicle, being localized only to ameloblasts and odontoblasts (Fig. 4h).

**Table 1:** Summary of immunostained proteins in the P1 molar tooth germ for the six genes under consideration

Tooth structure	Protein expression					
	POSTN ↓	TIMP2 ↓	MMP2 ↓	CK14 ↑	DSG3 ↑	PKP1 ↑
Dental follicle	**+**	**−**	**+**	**+**	**+**	**+**
Stellate reticulum	**−**	**−**	**+**	**+**	**−**	**−**
Stratum intermedium	**−**	**−**	**−**	**+**	**−**	**−**
Inner enamel epithelium	**−**	**−**	**−**	**+**	**−**	**+**
Ameloblast	**−**	**+**	**−**	**+**	**+**	**+**
Enamel	**−**	**−**	**−**	**−**	**−**	**−**
Dentine	**−**	**−**	**−**	**−**	**−**	**−**
Odontoblast	**−**	**+**	**−**	**−**	**−**	**−**
Pulp	**−**	**−**	**−**	**−**	**−**	**−**

Over-expressed genes (indicated by up arrows) were all present in the dental follicle as were under-expressed genes (indicated by down arrows), with the exception of TIMP2

The dental follicle was the most over-represented structure to be stained

## Discussion

To date there are no published studies on the transcriptome characteristics of primary molar ankylosis. The present study addresses this knowledge gap and has uncovered 432 DE genes, representing 2.3% of the total genes expressed by the furcal bone. From these genes, biological pathways relating to inflammatory response and epithelial cell turnover were predicted to be affected in ankylosis. In contrast with animal models of ankylosis, these findings open up new gateways for studying the pathogenesis of human molar ankylosis.

With respect to methodology, RNA-seq was selected over microarrays for its advantage in providing direct digital readout of expressed genes without reliance on pre-existing gene knowledge. Compared to hybridization approaches, RNA-seq has low background signals (from DNA contamination) and greater sensitivity for discovering low copy transcripts.^19^ Most importantly, RNA-seq is highly accurate for quantifying transcript abundance across technical and biological replicates, without the need for subsequent verification using quantitative PCR.^20^

To maximize signal strength, the experimental design needed to minimize clinical differences between the two sample groups. For this reason, molars with successors were excluded to prevent contaminating genes originating from the erupting tooth follicle. The two sample groups were designed to be as similar as possible, differentiated only by the presence or absence of infraocclusion without presumption of ankylosis. This is because studies have demonstrated ankylosis to be invariably present in infraoccluded primary molars with missing successors,^16^ but the same cannot be said for non-infraoccluded primary molars. Although the vast majority of the latter are non-ankylosed, histological ankylosis has occasionally been found.^21^ Therefore, non-infraoccluded teeth cannot be assumed to be non-ankylosed, especially since normal percussion sound and normality mobility do not reliably exclude ankylosis.^22^ In the current study, tissue scarcity precluded a parallel histological analysis, although even this would be of limited value given that histology cannot detect transcriptional activity leading to future clinical ankylosis.

The results showed that primary molar infraocclusion may be regarded as a phenotype with a molecular correlate involving at least 432 DE genes. However, since non-infraocclusion may represent a temporary absence of ankylosis in a dynamic disease, the differential gene outputs between the two groups may be viewed as a continuum, rather than as a distinct separation, between two different states of the pathology.

The above caveat notwithstanding, the pattern of gene expression was clearly different between the two groups, as demonstrated by hierarchical clustering (Fig. 1b) and PCA (Fig. 1c). Not only was there a clear separation between infraocclusion and non-infraocclusion, but both hierarchical clustering and PCA showed greater variation in the infraoccluded group compared to the non-infraoccluded group, which showed tighter clustering. This variation is a common finding in pathological conditions, since the pathology is often due to variable dysregulation of normally tightly regulated biological functions. For this reason, infraoccluded samples that looked like outliers (e.g. sample D5 in Figs. 1b, c) were not excluded, as they may represent the biological variability of ankylosis.

The number of DE genes (432) between the two groups represented only a small proportion (2.3%) of the total number of genes detected by RNA-seq. This small number appeared to reflect a strong degree of biological and molecular homogeneity between the two conditions (i.e. infraoccluded versus non-infraoccluded). In other transcriptome-wide studies using RNA-seq, for example comparing various types of cancers, the percentage of DE genes usually ranged from 30–70%, which reflects divergent tissue types with heterogeneous gene outputs.^23^ In contrast, exposure of a single cell type (*Acinetobacter baumannii*) to a single provocation (ethanol) elicited only 2% DE genes with RNA-seq.^24^ This supports the view that the current experimental design has maximized biological similarities between the sample groups, with the main difference being the presence, or not, of infraocclusion. It follows that the DE genes are likely to represent gene outputs associated with the infraocclusion phenotype.

Tooth ankylosis is a disruption in tooth eruption associated with loss of periodontal ligament (PDL) integrity. Given that genes causing disease are likely to be ontogenetically active during organ development,^25^ we reasoned that DE genes should also be expressed in developing tooth structures. Using immunohistochemical validation, we showed that all selected proteins were expressed in developing tooth structures. Although the spatial distribution of these proteins cannot speak to their etiology in tooth ankylosis, it would reaffirm the notion that pathological conditions frequently recapitulate their ontogenetic history during development. Of special note is the dental follicle (immunostained in five out of six proteins) that is comprised of pluripotent ectomesenchymal cells that subsequently give rise to the periodontal ligament, bone and cementum; all of which are involved in the ankylotic process. The detection of CK14 and PKP1 in the inner enamel epithelium deserves special mention given its developmental role leading to the epithelial rests of Malassez (ERM), a structure that has been implicated in ankylotic pathogenesis. The ERM play an important role in maintaining PDL space to prevent ankylosis.^26^ For example, experimental reduction of ERM distribution in rat teeth via denervation of the inferior alveolar nerve resulted in narrowing of the PDL space and ankylosis after 6 weeks. Subsequently, regeneration of ERM 10 weeks after denervation corresponded with widening of the PDL space.^27^ Other studies have observed that absence of the ERM in regenerated PDL is associated with narrower PDL space,^28^ and ERM are always present in the vital PDL areas of replanted teeth.^29^ These findings suggest that normal, so-called “quiescent” ERM may be involved in the maintenance of PDL space, and consequently, prevention of ankylosis. Therefore, ERM dysregulation may result in loss of its ability to prevent ankylosis.

Familial studies suggest that ankylosis is not a monogenic disease.^5^ Likewise, the present study did not identify a dominant gene for ankylosis. A pathological condition such as ankylosis requires the coordinated expression of multiple genes, often linked across multiple signalling pathways, as revealed in the current study. For this reason, the differential expression of one gene alone would be neither expected nor meaningful. On the other hand, the differential expression of multiple genes involved in a common biological pathway provides confidence that this pathway is being affected in infraocclusion. Pathway analysis using GSEA showed that certain pathways, in particular those associated with epithelial cell development and the inflammatory response, were over-represented in the infraoccluded group. Parallel analysis using IPA confirmed an increased activation of the inflammatory response and increased epithelial cell proliferation and differentiation in the infraoccluded group.

On closer inspection of the genes supporting epithelial change, keratins in particular were among the most highly expressed genes. Keratins form the intermediate filaments of epithelial cells and are known to reflect the tissue type as well as changes in differentiation states. The keratins in this study were consistent with those expressed in human ERM, and in particular, ERM undergoing proliferation and differentiation in response to inflammation^30–33^ (Table 2). The increased expression of desmosomal cadherins, desmoglein 2 (DSG2) and desmocollin 2 (DSC2), and the cytoplasmic desmosome-associated protein, plakophilin 1 (PKP1), are likewise consistent with human ERM.^32^ Collectively, the results suggest that there is a change in the ERM state during ankylosis, which may be associated with an increased inflammatory response.

**Table 2:** Differentially expressed keratins and their associated ERM states

Keratin	LogFC	ERM state
16	7.61	Normal^30^
6A	7.17	Normal^30^
13	6.06	Inflammatory^33^
14	5.94	Normal^30,32^ Increased when inflammatory^33^
5	5.88	Normal^30,32,33^
15	4.65	Normal^32^
19	3.86	Normal^30,32,33^
1	2.89	Normal^30^
4	N/A	Inflammatory (lower expression than Keratin 13)^33^

The above is concordant with established evidence that ankylosis is highly associated with root resorption, which is driven by pro-inflammatory mediators.^34,35^ Ankylosis in primary molars appears as an area of previous root resorption repaired by bone extending from the surrounding alveolus.^16,21^ In this way, ankylotic sites are constantly remodelling and relocating, closely following the progression of root resorption in an apical to cervical direction.^16^ Interestingly, root resorption, and presumably the accompanying inflammation, is greater in ankylosed, compared to non-ankylosed, primary teeth,^36–38^ further highlighting an association between ankylosis and inflammation.

The ebb and flow of the ankylotic process is reflected in multiple niches of hard tissue resorption and remineralization. Thus it was interesting to note that several mineralization-associated genes were found to be differentially expressed. These include decreased expression of bone-degrading genes (e.g. MMP2, MMP14^39^), increased expression of pro-mineralization genes (e.g. C4orf26,^40^ ODAM,^41,42^ BMP5, ASPN^43^), and decreased expression of anti-mineralization genes (POSTN,^44^ AXIN2,^45^ NFATC2^46^). A study of mutant mice bred for elevated Wnt signalling via β-catenin stabilization in osteocytes and cementocytes showed PDL space mineralization, failure of eruption, and dental ankylosis.^12^ In this study, it is possible that the decreased expression of AXIN2, which negatively regulates Wnt signalling via β-catenin degradation, similarly acts to increase Wnt signalling.^45^ Given that the differential expression pattern of these mineralization-associated genes seems to indicate a net anabolic effect, ankylosis may be associated with dysregulation of mineralization, as has been observed in animal models.^12–14^

Due to the case-control nature of this study, the results can only demonstrate an association, not a causal relationship, between ankylosis and the list of DE genes. The DE genes may well be the result, rather than the cause, of ankylosis, given that the condition was pre-existing before tissue sampling. Despite this limitation, the results of the present study would be valuable to future studies. This is because the behaviour of human primary molar ankylosis is currently not replicable in animal models, and any histological or transcriptomic analysis of human teeth precludes that tooth from longitudinal follow-up. Therefore, the aim of the study was to establish the differences in gene expression of ankylosed teeth, from which to build future experiments to explore a causal relationship, which could involve testing candidate genes in animal models, or modulating pathways highlighted to be important in ankylosis.

In conclusion, this case-control study found that primary molar ankylosis with infraocclusion was characterized by the differential expression of genes consistent with an increased inflammatory response and increased epithelial cell proliferation and differentiation. Independent validation of six DE genes showed their protein expression in developing tooth structures, especially the dental follicle. A hypothesis was generated that an increased inflammatory response and ERM dysregulation form part of the pathogenesis of primary molar ankylosis. This provides the foundation for hypothesis testing in future experimental models.

## Materials and methods

### Sample recruitment

Ethics approval was obtained from the University of Melbourne Human Research Ethics Committee (HREC ID: 1442486.1). Samples were donated, after written informed consent, by parents of children attending orthodontic clinics in Melbourne, Australia between December 2015 and January 2017. The inclusion criteria required that (1) all subjects were 15 years or under, medically healthy, and had not commenced orthodontic treatment to avoid confounders related to age, medical conditions, and orthodontic tooth movement, and (2) all primary molars were missing successors to avoid confounders emanating from erupting tooth follicles.

The infraocclusion group consisted of five subjects (three females and two males) with a mean age of 13.5 years (range 11.8–15.0 years), providing seven infraoccluded mandibular primary second molars. The non-infraocclusion group (controls) consisted of eight subjects (six females and two males) with a mean age of 11.5 years (range 7.9–3.8 years), providing nine non-infraoccluded mandibular primary second molars. This study followed the STROBE guidelines for human observational studies.

### Definition of infraocclusion

In this study, a tooth was deemed to be infraoccluded when the entire occlusal surface of the primary second molar was positioned at least 1 mm below the occlusal surface of the adjacent non-infraoccluded permanent molar, as judged by clinical and radiographic examination.^1^ Non-infraocclusion was defined as the entire occlusal surface of the primary second molar being level with the occlusal surface of the adjacent non-infraoccluded permanent molar. Additionally, there must be no vertical deficiency of the interdental alveolar bone on the panoramic radiograph.

### Sample preparation and RNA-seq

The steps of the experimental protocol are illustrated in Supplementary Fig. 1. Bone was harvested at time of extraction from the bifurcation area of mandibular primary second molars, quickly rinsed in sterile phosphate-buffered saline, then immediately stored in RNA*later*^®^ (Qiagen, Switzerland) following manufacturer’s instructions. Samples were homogenized using a Bullet Blender^®^ (Next Advance, USA) and 1.5 mL Navy Eppendorf^®^ Bead Lysis microtubes (Next Advance, USA) filled with six additional 3.5 mm stainless steel UFO beads (Next Advance, USA), following the protocol described by Carter, et al.,^47^ with a modification to increase the blending time to 8 min at speed 12.

Total RNA was isolated using the RNeasy™ Mini Kit (Qiagen, Switzerland) following manufacturer’s instructions. The RNA yield, RNA quality, and salt contamination of all samples were measured using the Bioanalyzer (Agilent Technologies, USA) and NanoDrop™ spectrophotometer (Thermo Fisher Scientific, USA). Library preparation involved removal of rRNA using Ribo-Zero™ Gold (Illumina, USA) and conversion to cDNA. Raw 100 bp single-end RNA-seq data was produced using the Illumina HiSeq 2500 system (Illumina, USA).

### Bioinformatic analysis

The sequence reads were aligned against the human genome (Build version HG38) using the Tophat aligner (v.2.0.14),^48^ utilizing the reference annotation based transcript (RABT) assembly option. Transcripts were assembled with the Stringtie tool (v1.2.4).^49^

The read counts were used to estimate gene expression and identify DE genes using the R package (R version 3.2.0) ‘edgeR’ (v.3.10.5).^50^ Genes with >10 counts in at least seven samples (i.e. number of biological replicates) were retained for further analysis. The normalization method was RUV (remove unwanted variation).^51^ All genes were included to compute the factor of unwanted variation using the function RUVr. The *p*-value was adjusted for multiple testing by using the Benjamini-Hochberg correction to obtain the FDR.

Hierarchical clustering and the resulting heatmap were computed using all 432 DE genes with FDR < 0.05. The normalized read counts were scaled (mean-centred and divided by standard deviation), and the distance metric was Euclidean. PCA was computed using all identified genes to visualize transcriptome variation between samples.

### Pathway analysis

DE genes were further studied to define their cellular and biological significance. GSEA was performed against all GO gene sets in the molecular signatures database (v6.0).^52^ Next, to map significant gene sets (FDR < 0.05) and their overlap (overlap coefficient > 0.25), we used Cytoscape (v3.5.1) software^53^ with the EnrichmentMap plugin.^54^

The input list of DE genes for IPA (Qiagen, Switzerland) (accessed May 2017) had significance cut-offs of FDR < 0.05 and logFC ≤−0.59 or ≥0.59 (equivalent to a 1.5-fold change or more).

### Immunohistochemistry

Immunohistochemistry was carried out on coronal sections of mandibles containing developing mouse tooth germs from postnatal day 1 (P1) mice and immersed in 4% paraformaldehyde at 4 °C for 24 h. Following embedding in paraffin, serial sections (5 µm thick) were obtained for immunohistochemistry and staining with haematoxylin and eosin. For immunofluorescence staining, sections were deparaffinised with xylene, followed by rehydration through graded alcohols and distilled water. Antigen retrieval was carried out by boiling the sections in sodium citrate buffer for 10 min. Sections were then washed with 0.1% PBS with Triton-X and blocked with 10% normal horse serum for 1 h at room temperature. Sections were incubated with primary antibodies overnight followed by appropriate secondary antibodies for 1 h at room temperature, or stained with DAPI (1:10 000, Dako, Carpinteria, CA) before mounting under glass cover slips with anti-fade mounting reagent.

Primary antibodies were: rabbit polyclonal anti-periostin (POSTN) (1:100, LifeSpan Biosciences, Seattle), mouse monoclonal anti-tissue inhibitor of metalloproteinases 2 (TIMP2) (1:400, Millipore, Temecula), rabbit polyclonal anti-matrix metalloproteinase 2 (MMP2) (1:50, Millipore, Temecula), mouse monoclonal anti-cytokeratin 14 (CK14) (1:200, Thermo Fisher Scientific, Rockford), mouse monoclonal anti-desmoglein 3 (DSG3) (1:100, Thermo Fisher Scientific, Rockford) and mouse monoclonal anti-plakophilin 1 (PKP1) (1:50, Abcam, Cambridge). Secondary antibodies were: Alexa Fluor 488-conjugated goat anti-mouse IgG (1:500, Molecular Probes) and Alexa Fluor 488-conjugated goat anti-rabbit IgG (1:500, Molecular Probes). Imaging for immunofluorescence was performed on a Zeiss 780 Confocal Microscope.

## Supplementary information

Supplementary Tables and Figures

## Data Availability

The dataset generated by this study is publicly archived in the NIH Sequence Read Archive (accession number PRJNA558843).
