# Supplementary material for: Transcriptome analysis of ankylosed primary molars with infraocclusion
Source: Int J Oral Sci. 2020 Feb 21;12:7. doi: 10.1038/s41368-019-0070-1 (PMC7033215; doi:10.1038/s41368-019-0070-1)
Supplement: Supplementary file 1 — Supplementary Tables and Figures [file 41368_2019_70_MOESM1_ESM.docx]

Transcriptome analysis of ankylosed primary molars with infraocclusion

Annie Tong^1^, Yuh-Lit Chow^2^, Katie Xu^1^, Rita Hardiman^1^, Paul Schneider^1^, Seong-Seng Tan^2^

^1^ Melbourne Dental School, The University of Melbourne, Australia

^2^ Florey Institute of Neuroscience, The University of Melbourne, Australia

*Supplementary files*


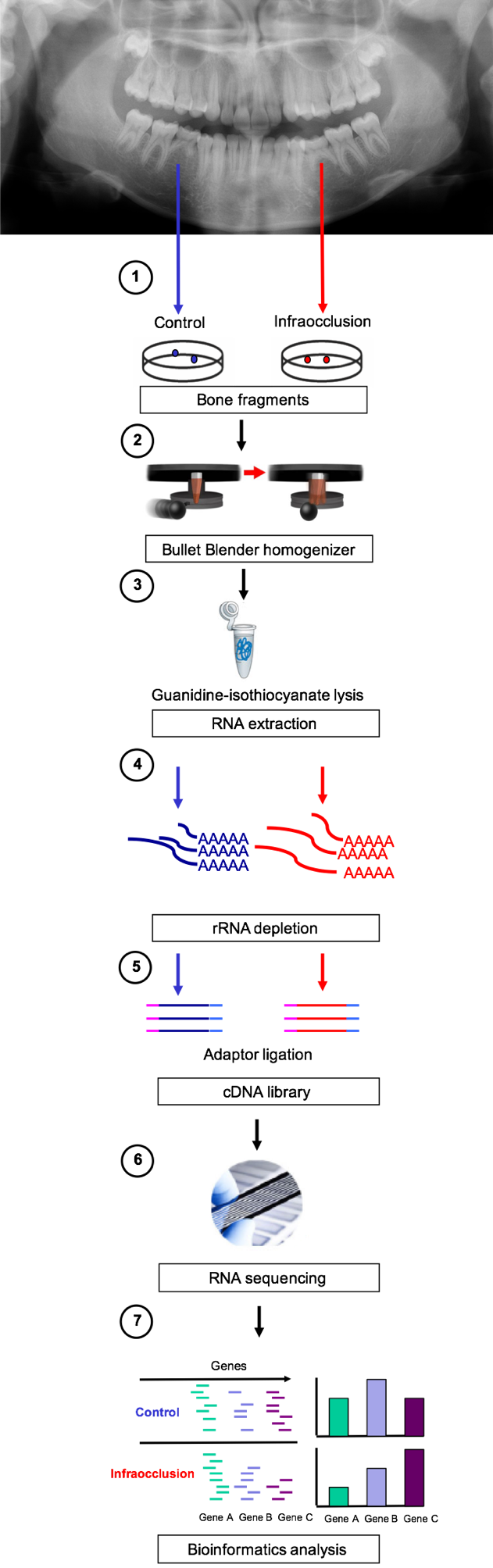
**Supplementary Figure 1.** Workflow from tissue harvest to bioinformatics analysis

**Supplementary Table 1.** DE genes (432) in infraocclusion (FDR <0.05 ranked by log_2_ fold change [logFC])

| Gene Symbol | Gene Full Name | logFC | FDR |
| --- | --- | --- | --- |
| KRT16 | keratin 16 | 7.61 | 0.00008 |
| KRT6A | keratin 6A | 7.17 | 0.00017 |
| C4orf26 | chromosome 4 open reading frame 26 | 6.41 | 0.00022 |
| SPRR2E | small proline rich protein 2E | 6.18 | 0.00228 |
| TMPRSS11D | transmembrane protease, serine 11D | 6.18 | 0.00027 |
| A2ML1 | alpha-2-macroglobulin like 1 | 6.10 | 0.00032 |
| KRT13 | keratin 13 | 6.06 | 0.00067 |
| TMPRSS11A | transmembrane protease, serine 11A | 6.06 | 0.00078 |
| KRT14 | keratin 14 | 5.94 | 0.00021 |
| KRT5 | keratin 5 | 5.88 | 0.00014 |
| RHCG | Rh family C glycoprotein | 5.84 | 0.00065 |
| CRNN | cornulin | 5.82 | 0.00247 |
| DSG3 | desmoglein 3 | 5.77 | 0.00031 |
| SPRR1B | small proline rich protein 1B | 5.74 | 0.00539 |
| DSC3 | desmocollin 3 | 5.56 | 0.00020 |
| SPRR3 | small proline rich protein 3 | 5.53 | 0.00228 |
| SPINK5 | serine peptidase inhibitor, Kazal type 5 | 5.52 | 0.00039 |
| ODAM | odontogenic, ameloblast asssociated | 5.42 | 0.00047 |
| SPRR2A | small proline rich protein 2A | 5.40 | 0.00156 |
| EHF | ETS homologous factor | 5.31 | 0.00022 |
| GBP6 | guanylate binding protein family member 6 | 5.25 | 0.00022 |
| SFN | stratifin | 5.21 | 0.00031 |
| SLPI | secretory leukocyte peptidase inhibitor | 5.20 | 0.00014 |
| CLCA2 | chloride channel accessory 2 | 5.20 | 0.00001 |
| FDCSP | follicular dendritic cell secreted protein | 5.13 | 0.00276 |
| GJB2 | gap junction protein beta 2 | 4.94 | 0.00065 |
| CSTA | cystatin A | 4.86 | 0.00011 |
| MMP12 | matrix metallopeptidase 12 | 4.85 | 0.00102 |
| SLC5A1 | solute carrier family 5 member 1 | 4.80 | 0.00064 |
| SERPINB5 | serpin family B member 5 | 4.80 | 0.00228 |
| SCEL | sciellin | 4.79 | 0.00176 |
| PERP | PERP, TP53 apoptosis effector | 4.78 | 0.00010 |
| LAMC2 | laminin subunit gamma 2 | 4.75 | 0.00092 |
| S100A2 | S100 calcium binding protein A2 | 4.74 | 0.00170 |
| ADAMTS20 | ADAM metallopeptidase with thrombospondin type 1 motif 20 | 4.71 | 0.00067 |
| KRT15 | keratin 15 | 4.65 | 0.00080 |
| TRIM29 | tripartite motif containing 29 | 4.64 | 0.00078 |
| TACSTD2 | tumor-associated calcium signal transducer 2 | 4.59 | 0.00067 |
| MIR205HG | - | 4.59 | 0.00128 |
| DSP | desmoplakin | 4.52 | 0.00023 |
| CDH3 | cadherin 3 | 4.50 | 0.00032 |
| FAM83B | family with sequence similarity 83 member B | 4.48 | 0.00079 |
| MAL | mal, T-cell differentiation protein | 4.42 | 0.00067 |
| S100A8 | S100 calcium binding protein A8 | 4.38 | 0.00016 |
| COL17A1 | collagen type XVII alpha 1 chain | 4.35 | 0.00026 |
| CXCL1 | C-X-C motif chemokine ligand 1 | 4.27 | 0.00352 |
| PKP1 | plakophilin 1 | 4.26 | 0.00031 |
| CLDN1 | claudin 1 | 4.21 | 0.00052 |
| LAD1 | ladinin 1 | 4.18 | 0.00117 |
| IL1RN | interleukin 1 receptor antagonist | 4.02 | 0.00160 |
| IL20RB | interleukin 20 receptor subunit beta | 3.99 | 0.00041 |
| ANXA8 | annexin A8 | 3.97 | 0.00115 |
| ADH1B | alcohol dehydrogenase 1B (class I), beta polypeptide | 3.97 | 0.00037 |
| IRF6 | interferon regulatory factor 6 | 3.96 | 0.00066 |
| DMKN | dermokine | 3.95 | 0.00243 |
| TMPRSS2 | transmembrane protease, serine 2 | 3.93 | 0.00228 |
| XDH | xanthine dehydrogenase | 3.89 | 0.00111 |
| SLC7A11 | solute carrier family 7 member 11 | 3.88 | 0.00032 |
| TNS4 | tensin 4 | 3.88 | 0.00488 |
| KRT19 | keratin 19 | 3.86 | 0.00159 |
| HBD | hemoglobin subunit delta | 3.85 | 0.01376 |
| CDH1 | cadherin 1 | 3.83 | 0.00031 |
| S100A9 | S100 calcium binding protein A9 | 3.82 | 0.00031 |
| ESRP1 | epithelial splicing regulatory protein 1 | 3.81 | 0.00602 |
| CLCA4 | chloride channel accessory 4 | 3.81 | 0.01297 |
| LCN2 | lipocalin 2 | 3.79 | 0.00218 |
| LAMB3 | laminin subunit beta 3 | 3.78 | 0.00022 |
| BBOX1 | gamma-butyrobetaine hydroxylase 1 | 3.77 | 0.00023 |
| CERS3 | ceramide synthase 3 | 3.75 | 0.00222 |
| FAT2 | FAT atypical cadherin 2 | 3.71 | 0.00293 |
| TMPRSS4 | transmembrane protease, serine 4 | 3.59 | 0.00189 |
| LYPD3 | LY6/PLAUR domain containing 3 | 3.59 | 0.00085 |
| FAM110C | family with sequence similarity 110 member C | 3.58 | 0.00032 |
| FMO1 | flavin containing monooxygenase 1 | 3.53 | 0.00767 |
| SLC26A9 | solute carrier family 26 member 9 | 3.47 | 0.00841 |
| GRHL1 | grainyhead like transcription factor 1 | 3.47 | 0.00078 |
| ITGB6 | integrin subunit beta 6 | 3.45 | 0.00040 |
| EVPL | envoplakin | 3.44 | 0.00034 |
| AQP3 | aquaporin 3 (Gill blood group) | 3.43 | 0.00105 |
| CA1 | carbonic anhydrase 1 | 3.41 | 0.00539 |
| MACC1 | MACC1, MET transcriptional regulator | 3.39 | 0.00152 |
| ABCA12 | ATP binding cassette subfamily A member 12 | 3.30 | 0.00606 |
| AHSP | alpha hemoglobin stabilizing protein | 3.26 | 0.00841 |
| MPZL2 | myelin protein zero like 2 | 3.20 | 0.00027 |
| PCK1 | phosphoenolpyruvate carboxykinase 1 | 3.19 | 0.00646 |
| DSC2 | desmocollin 2 | 3.16 | 0.00034 |
| PDZK1IP1 | PDZK1 interacting protein 1 | 3.15 | 0.00254 |
| NECTIN4 | nectin cell adhesion molecule 4 | 3.13 | 0.00293 |
| NECAB1 | N-terminal EF-hand calcium binding protein 1 | 3.13 | 0.00085 |
| HOPX | HOP homeobox | 3.12 | 0.00145 |
| GSDMC | gasdermin C | 3.07 | 0.01114 |
| DDIT4L | DNA damage inducible transcript 4 like | 3.07 | 0.00256 |
| BPI | bactericidal/permeability-increasing protein | 3.06 | 0.00691 |
| TGM1 | transglutaminase 1 | 3.02 | 0.00474 |
| LTF | lactotransferrin | 2.95 | 0.01428 |
| KRT1 | keratin 1 | 2.89 | 0.01036 |
| TMPRSS13 | transmembrane protease, serine 13 | 2.86 | 0.00579 |
| C1orf116 | chromosome 1 open reading frame 116 | 2.85 | 0.01434 |
| SLC9A3 | solute carrier family 9 member A3 | 2.84 | 0.00910 |
| HOOK1 | hook microtubule tethering protein 1 | 2.83 | 0.00101 |
| RGS4 | regulator of G-protein signaling 4 | 2.83 | 0.01147 |
| CDCP1 | CUB domain containing protein 1 | 2.80 | 0.00328 |
| PADI4 | peptidyl arginine deiminase 4 | 2.80 | 0.00425 |
| MLXIPL | MLX interacting protein like | 2.78 | 0.01631 |
| GPR88 | G protein-coupled receptor 88 | 2.76 | 0.00079 |
| PITX1 | paired like homeodomain 1 | 2.74 | 0.00593 |
| ALOX12 | arachidonate 12-lipoxygenase, 12S type | 2.74 | 0.01009 |
| BMP5 | bone morphogenetic protein 5 | 2.73 | 0.00081 |
| EPHA1 | EPH receptor A1 | 2.73 | 0.00951 |
| GPRC5A | G protein-coupled receptor class C group 5 member A | 2.71 | 0.00251 |
| PRRG4 | proline rich and Gla domain 4 | 2.71 | 0.00166 |
| FABP5P7 | - | 2.71 | 0.00159 |
| FMO2 | flavin containing monooxygenase 2 | 2.70 | 0.00034 |
| S100A12 | S100 calcium binding protein A12 | 2.70 | 0.00152 |
| FABP5 | fatty acid binding protein 5 | 2.70 | 0.00078 |
| CHRDL1 | chordin like 1 | 2.69 | 0.00004 |
| SPTA1 | spectrin alpha, erythrocytic 1 | 2.61 | 0.01309 |
| LAMA3 | laminin subunit alpha 3 | 2.60 | 0.00590 |
| MYH14 | myosin heavy chain 14 | 2.60 | 0.00348 |
| MYO5B | myosin VB | 2.59 | 0.00488 |
| PROM2 | prominin 2 | 2.58 | 0.00658 |
| KLF5 | Kruppel like factor 5 | 2.57 | 0.00485 |
| KRT16P6 | - | 2.57 | 0.02361 |
| SPTBN2 | spectrin beta, non-erythrocytic 2 | 2.56 | 0.00445 |
| PLIN1 | perilipin 1 | 2.55 | 0.03616 |
| SPINT2 | serine peptidase inhibitor, Kunitz type 2 | 2.53 | 0.00196 |
| PLS1 | plastin 1 | 2.49 | 0.00604 |
| CLDN7 | claudin 7 | 2.49 | 0.02029 |
| OSGIN1 | oxidative stress induced growth inhibitor 1 | 2.49 | 0.00492 |
| TTC22 | tetratricopeptide repeat domain 22 | 2.48 | 0.01178 |
| LVRN | laeverin | 2.47 | 0.01161 |
| CDS1 | CDP-diacylglycerol synthase 1 | 2.46 | 0.01494 |
| PITX2 | paired like homeodomain 2 | 2.45 | 0.01468 |
| CAPNS2 | calpain small subunit 2 | 2.41 | 0.01464 |
| ALDH1L1 | aldehyde dehydrogenase 1 family member L1 | 2.41 | 0.00196 |
| TP63 | tumor protein p63 | 2.39 | 0.01428 |
| CEACAM1 | carcinoembryonic antigen related cell adhesion molecule 1 | 2.37 | 0.00036 |
| VLDLR | very low density lipoprotein receptor | 2.37 | 0.00485 |
| FABP4 | fatty acid binding protein 4 | 2.36 | 0.01800 |
| GPD1 | glycerol-3-phosphate dehydrogenase 1 | 2.35 | 0.04287 |
| FERMT1 | fermitin family member 1 | 2.35 | 0.02196 |
| ST14 | suppression of tumorigenicity 14 | 2.32 | 0.00293 |
| ATG9B | autophagy related 9B | 2.31 | 0.04228 |
| AQP7 | aquaporin 7 | 2.31 | 0.00321 |
| AZGP1 | alpha-2-glycoprotein 1, zinc-binding | 2.28 | 0.01611 |
| MGST1 | microsomal glutathione S-transferase 1 | 2.28 | 0.00038 |
| SLC19A3 | solute carrier family 19 member 3 | 2.24 | 0.00058 |
| S100P | S100 calcium binding protein P | 2.20 | 0.03153 |
| ABCA13 | ATP binding cassette subfamily A member 13 | 2.19 | 0.00387 |
| BNIPL | BCL2 interacting protein like | 2.17 | 0.02202 |
| APOBEC3A | apolipoprotein B mRNA editing enzyme catalytic subunit 3A | 2.16 | 0.01766 |
| JCHAIN | joining chain of multimeric IgA and IgM | 2.15 | 0.04734 |
| C3 | complement C3 | 2.14 | 0.00031 |
| ANXA3 | annexin A3 | 2.13 | 0.00335 |
| PLEKHG6 | pleckstrin homology and RhoGEF domain containing G6 | 2.13 | 0.00160 |
| RP11-544L8__B.4 | - | 2.13 | 0.00826 |
| GCLC | glutamate-cysteine ligase catalytic subunit | 2.13 | 0.00247 |
| STOX1 | storkhead box 1 | 2.13 | 0.00001 |
| ZNF185 | zinc finger protein 185 (LIM domain) | 2.11 | 0.00758 |
| FAM196B | family with sequence similarity 196 member B | 2.11 | 0.00247 |
| LIPE | lipase E, hormone sensitive type | 2.10 | 0.00294 |
| CDHR1 | cadherin related family member 1 | 2.06 | 0.02980 |
| MAP3K9 | mitogen-activated protein kinase kinase kinase 9 | 2.06 | 0.01434 |
| ADD2 | adducin 2 | 2.03 | 0.01502 |
| TGFA | transforming growth factor alpha | 2.02 | 0.00445 |
| G0S2 | G0/G1 switch 2 | 2.02 | 0.01297 |
| HTR7 | 5-hydroxytryptamine receptor 7 | 2.00 | 0.00290 |
| TPD52L1 | tumor protein D52-like 1 | 1.98 | 0.00368 |
| PRLR | prolactin receptor | 1.97 | 0.00417 |
| RBP1 | retinol binding protein 1 | 1.96 | 0.00433 |
| PAQR5 | progestin and adipoQ receptor family member 5 | 1.96 | 0.02029 |
| FAM83F | family with sequence similarity 83 member F | 1.94 | 0.01494 |
| ACVR1C | activin A receptor type 1C | 1.92 | 0.00196 |
| NPR3 | natriuretic peptide receptor 3 | 1.91 | 0.01448 |
| SHISA6 | shisa family member 6 | 1.89 | 0.01297 |
| ANXA8L1 | annexin A8-like 1 | 1.89 | 0.01547 |
| SPINT1 | serine peptidase inhibitor, Kunitz type 1 | 1.88 | 0.02520 |
| RASSF10 | Ras association domain family member 10 | 1.87 | 0.03752 |
| OR51E1 | olfactory receptor family 51 subfamily E member 1 | 1.84 | 0.00547 |
| PFKFB1 | 6-phosphofructo-2-kinase/fructose-2,6-biphosphatase 1 | 1.82 | 0.01376 |
| ANGPTL4 | angiopoietin like 4 | 1.81 | 0.00031 |
| IL18 | interleukin 18 | 1.80 | 0.00869 |
| STAB2 | stabilin 2 | 1.78 | 0.03090 |
| F2RL1 | F2R like trypsin receptor 1 | 1.78 | 0.00910 |
| RP11-38H17.1 | - | 1.77 | 0.00388 |
| TUBA4A | tubulin alpha 4a | 1.74 | 0.02420 |
| CDA | cytidine deaminase | 1.73 | 0.03281 |
| SV2B | synaptic vesicle glycoprotein 2B | 1.69 | 0.02234 |
| SYTL1 | synaptotagmin like 1 | 1.69 | 0.03512 |
| CALN1 | calneuron 1 | 1.68 | 0.01670 |
| DHRS9 | dehydrogenase/reductase 9 | 1.66 | 0.02437 |
| DSG2 | desmoglein 2 | 1.65 | 0.00696 |
| ST6GALNAC2 | ST6 N-acetylgalactosaminide alpha-2,6-sialyltransferase 2 | 1.64 | 0.01115 |
| GPR182 | G protein-coupled receptor 182 | 1.61 | 0.01376 |
| CXADR | coxsackie virus and adenovirus receptor | 1.60 | 0.00445 |
| PKP2 | plakophilin 2 | 1.60 | 0.01434 |
| TNFSF10 | tumor necrosis factor superfamily member 10 | 1.57 | 0.00474 |
| FCGR3A | Fc fragment of IgG receptor IIIa | 1.54 | 0.01241 |
| TNFRSF21 | TNF receptor superfamily member 21 | 1.54 | 0.03354 |
| IMPA2 | inositol monophosphatase 2 | 1.53 | 0.00445 |
| PRSS12 | protease, serine 12 | 1.53 | 0.02361 |
| SLC9A3R1 | SLC9A3 regulator 1 | 1.52 | 0.04297 |
| LURAP1L | leucine rich adaptor protein 1 like | 1.50 | 0.00022 |
| RP11-392P7.6 | - | 1.48 | 0.02266 |
| PDK4 | pyruvate dehydrogenase kinase 4 | 1.46 | 0.00004 |
| CD80 | CD80 molecule | 1.45 | 0.00255 |
| KLF15 | Kruppel like factor 15 | 1.43 | 0.01147 |
| TMEM154 | transmembrane protein 154 | 1.42 | 0.03402 |
| ARAP2 | ArfGAP with RhoGAP domain, ankyrin repeat and PH domain 2 | 1.42 | 0.02641 |
| KLRF1 | killer cell lectin like receptor F1 | 1.40 | 0.01139 |
| LINC01239 | - | 1.40 | 0.02641 |
| SH2D1B | SH2 domain containing 1B | 1.39 | 0.01940 |
| CHI3L2 | chitinase 3 like 2 | 1.38 | 0.02123 |
| IKZF2 | IKAROS family zinc finger 2 | 1.37 | 0.00175 |
| KIAA0895 | KIAA0895 | 1.37 | 0.00957 |
| ACTR3C | ARP3 actin-related protein 3 homolog C | 1.36 | 0.00815 |
| SLC24A3 | solute carrier family 24 member 3 | 1.36 | 0.02436 |
| CLEC2B | C-type lectin domain family 2 member B | 1.35 | 0.02102 |
| NCF1B | - | 1.34 | 0.03437 |
| RALGPS2 | Ral GEF with PH domain and SH3 binding motif 2 | 1.34 | 0.01590 |
| GPX3 | glutathione peroxidase 3 | 1.32 | 0.01739 |
| F11R | F11 receptor | 1.30 | 0.02741 |
| ADRB2 | adrenoceptor beta 2 | 1.30 | 0.03906 |
| S100B | S100 calcium binding protein B | 1.29 | 0.00485 |
| SIGLEC14 | sialic acid binding Ig like lectin 14 | 1.28 | 0.04433 |
| VNN1 | vanin 1 | 1.27 | 0.02083 |
| MPZL3 | myelin protein zero like 3 | 1.26 | 0.03573 |
| PARD6B | par-6 family cell polarity regulator beta | 1.25 | 0.01424 |
| PYGL | glycogen phosphorylase L | 1.25 | 0.00254 |
| ADH1C | alcohol dehydrogenase 1C (class I), gamma polypeptide | 1.25 | 0.01618 |
| NSG1 | Neuron-specific protein family member 1 | 1.24 | 0.00613 |
| ABCA8 | ATP binding cassette subfamily A member 8 | 1.24 | 0.00258 |
| MARC1 | mitochondrial amidoxime reducing component 1 | 1.22 | 0.04108 |
| CYP4F12 | cytochrome P450 family 4 subfamily F member 12 | 1.21 | 0.02299 |
| MME | membrane metalloendopeptidase | 1.20 | 0.00727 |
| PDE8B | phosphodiesterase 8B | 1.20 | 0.00196 |
| LRRK2 | leucine rich repeat kinase 2 | 1.19 | 0.02234 |
| GBP1P1 | - | 1.18 | 0.03470 |
| TMEM51-AS1 | - | 1.18 | 0.00386 |
| ADGRG6 | adhesion G protein-coupled receptor G6 | 1.17 | 0.00940 |
| CHL1 | cell adhesion molecule L1 like | 1.15 | 0.02762 |
| SULT1B1 | sulfotransferase family 1B member 1 | 1.15 | 0.01669 |
| VAMP8 | vesicle associated membrane protein 8 | 1.14 | 0.03396 |
| DHRS3 | dehydrogenase/reductase 3 | 1.14 | 0.00292 |
| SORBS1 | sorbin and SH3 domain containing 1 | 1.13 | 0.03190 |
| ADAM28 | ADAM metallopeptidase domain 28 | 1.11 | 0.02238 |
| NIPSNAP3B | nipsnap homolog 3B | 1.11 | 0.04162 |
| FAM213A | family with sequence similarity 213 member A | 1.10 | 0.01147 |
| PLEKHA7 | pleckstrin homology domain containing A7 | 1.08 | 0.00377 |
| TM7SF2 | transmembrane 7 superfamily member 2 | 1.07 | 0.04424 |
| ZSCAN31 | zinc finger and SCAN domain containing 31 | 1.05 | 0.00091 |
| PLBD1 | phospholipase B domain containing 1 | 1.03 | 0.00499 |
| ALDH4A1 | aldehyde dehydrogenase 4 family member A1 | 1.02 | 0.02236 |
| PLAT | plasminogen activator, tissue type | 1.00 | 0.01201 |
| LRRC8D | leucine rich repeat containing 8 family member D | 0.98 | 0.04840 |
| FSIP2 | fibrous sheath interacting protein 2 | 0.98 | 0.04882 |
| SAMD9 | sterile alpha motif domain containing 9 | 0.97 | 0.02076 |
| NQO1 | NAD(P)H quinone dehydrogenase 1 | 0.95 | 0.02524 |
| TPRG1 | tumor protein p63 regulated 1 | 0.94 | 0.02324 |
| ALDH1A1 | aldehyde dehydrogenase 1 family member A1 | 0.93 | 0.00525 |
| PLPP3 | phospholipid phosphatase 3 | 0.93 | 0.01540 |
| CCL28 | C-C motif chemokine ligand 28 | 0.92 | 0.02762 |
| PTGER3 | prostaglandin E receptor 3 | 0.91 | 0.02234 |
| PAIP2B | poly(A) binding protein interacting protein 2B | 0.91 | 0.01310 |
| BHMT2 | betaine--homocysteine S-methyltransferase 2 | 0.90 | 0.04941 |
| KLKB1 | kallikrein B1 | 0.89 | 0.04125 |
| ABHD5 | abhydrolase domain containing 5 | 0.86 | 0.02361 |
| ACER2 | alkaline ceramidase 2 | 0.85 | 0.03313 |
| GABRE | gamma-aminobutyric acid type A receptor epsilon subunit | 0.84 | 0.04424 |
| USP53 | ubiquitin specific peptidase 53 | 0.82 | 0.01946 |
| DDX12P | - | 0.82 | 0.02234 |
| FRRS1 | ferric chelate reductase 1 | 0.80 | 0.01202 |
| PDCD1LG2 | programmed cell death 1 ligand 2 | 0.80 | 0.02835 |
| PPIP5K1 | diphosphoinositol pentakisphosphate kinase 1 | 0.79 | 0.00628 |
| VAV3 | vav guanine nucleotide exchange factor 3 | 0.76 | 0.01631 |
| FGD4 | FYVE, RhoGEF and PH domain containing 4 | 0.72 | 0.02254 |
| SLFN13 | schlafen family member 13 | 0.71 | 0.02420 |
| TDRD7 | tudor domain containing 7 | 0.69 | 0.02541 |
| TLR5 | toll like receptor 5 | 0.68 | 0.01672 |
| OGFRL1 | opioid growth factor receptor like 1 | 0.67 | 0.03612 |
| ZNF204P | - | 0.67 | 0.02591 |
| CEP152 | centrosomal protein 152 | 0.64 | 0.04424 |
| RP11-701H24.4 | - | 0.63 | 0.02254 |
| PPM1L | protein phosphatase, Mg2+/Mn2+ dependent 1L | 0.60 | 0.03117 |
| FIG4 | FIG4 phosphoinositide 5-phosphatase | 0.52 | 0.03664 |
| MMP14 | matrix metallopeptidase 14 | -0.57 | 0.04185 |
| MARVELD1 | MARVEL domain containing 1 | -0.63 | 0.02769 |
| SIK3 | SIK family kinase 3 | -0.65 | 0.04119 |
| CRY1 | cryptochrome circadian clock 1 | -0.67 | 0.01040 |
| SAMD4A | sterile alpha motif domain containing 4A | -0.67 | 0.04840 |
| TIMP2 | TIMP metallopeptidase inhibitor 2 | -0.68 | 0.04108 |
| GJC1 | gap junction protein gamma 1 | -0.68 | 0.03874 |
| CAMK1D | calcium/calmodulin dependent protein kinase ID | -0.70 | 0.02234 |
| P3H3 | prolyl 3-hydroxylase 3 | -0.72 | 0.02651 |
| ADAMTS4 | ADAM metallopeptidase with thrombospondin type 1 motif 4 | -0.72 | 0.02536 |
| CHRD | chordin | -0.73 | 0.04941 |
| C3orf70 | chromosome 3 open reading frame 70 | -0.76 | 0.02278 |
| SH3PXD2A | SH3 and PX domains 2A | -0.76 | 0.01346 |
| CCDC3 | coiled-coil domain containing 3 | -0.76 | 0.03527 |
| LMNA | lamin A/C | -0.77 | 0.04901 |
| STK32B | serine/threonine kinase 32B | -0.77 | 0.01590 |
| ADAMTS2 | ADAM metallopeptidase with thrombospondin type 1 motif 2 | -0.80 | 0.01551 |
| KCNA2 | potassium voltage-gated channel subfamily A member 2 | -0.81 | 0.01040 |
| TRIM47 | tripartite motif containing 47 | -0.81 | 0.02347 |
| MMP2 | matrix metallopeptidase 2 | -0.81 | 0.02387 |
| NAT14 | N-acetyltransferase 14 (putative) | -0.81 | 0.02076 |
| PROB1 | proline rich basic protein 1 | -0.82 | 0.04424 |
| LGI4 | leucine rich repeat LGI family member 4 | -0.86 | 0.00386 |
| SLC7A2 | solute carrier family 7 member 2 | -0.86 | 0.00442 |
| MYADM | myeloid associated differentiation marker | -0.86 | 0.03470 |
| RORA | RAR related orphan receptor A | -0.87 | 0.02029 |
| ARHGEF19 | Rho guanine nucleotide exchange factor 19 | -0.88 | 0.03059 |
| HPX | hemopexin | -0.89 | 0.01914 |
| NXN | nucleoredoxin | -0.89 | 0.00978 |
| SDK2 | sidekick cell adhesion molecule 2 | -0.89 | 0.00583 |
| RP11-53B2.6 | - | -0.90 | 0.03742 |
| AQP1 | aquaporin 1 (Colton blood group) | -0.91 | 0.00575 |
| B4GALNT1 | beta-1,4-N-acetyl-galactosaminyltransferase 1 | -0.92 | 0.01352 |
| TOX2 | TOX high mobility group box family member 2 | -0.92 | 0.02278 |
| C18orf15 | - | -0.93 | 0.01468 |
| AXIN2 | axin 2 | -0.93 | 0.01111 |
| ZNF469 | zinc finger protein 469 | -0.94 | 0.04941 |
| COL5A1 | collagen type V alpha 1 chain | -0.96 | 0.01347 |
| CYS1 | cystin 1 | -0.96 | 0.01875 |
| COL6A2 | collagen type VI alpha 2 chain | -0.96 | 0.01331 |
| AGMAT | agmatinase | -0.98 | 0.04424 |
| FBXL7 | F-box and leucine rich repeat protein 7 | -0.99 | 0.00254 |
| TMIE | transmembrane inner ear | -1.00 | 0.02159 |
| EFCAB1 | EF-hand calcium binding domain 1 | -1.00 | 0.02060 |
| ARSJ | arylsulfatase family member J | -1.02 | 0.00766 |
| COL16A1 | collagen type XVI alpha 1 chain | -1.02 | 0.02215 |
| APLP1 | amyloid beta precursor like protein 1 | -1.02 | 0.00321 |
| POLR2J2 | RNA polymerase II subunit J2 | -1.02 | 0.02583 |
| OLFM2 | olfactomedin 2 | -1.03 | 0.03190 |
| RIPPLY2 | ripply transcriptional repressor 2 | -1.04 | 0.02254 |
| FLNC | filamin C | -1.06 | 0.02234 |
| RP11-339B21.8 | - | -1.07 | 0.04360 |
| PPFIA3 | PTPRF interacting protein alpha 3 | -1.07 | 0.00436 |
| GUCY1A2 | guanylate cyclase 1 soluble subunit alpha 2 | -1.07 | 0.00545 |
| MYCN | v-myc avian myelocytomatosis viral oncogene neuroblastoma derived homolog | -1.08 | 0.01672 |
| AVPR1A | arginine vasopressin receptor 1A | -1.08 | 0.01049 |
| MMP19 | matrix metallopeptidase 19 | -1.09 | 0.01160 |
| PLEKHG4 | pleckstrin homology and RhoGEF domain containing G4 | -1.10 | 0.01018 |
| KIF26B | kinesin family member 26B | -1.11 | 0.00591 |
| RP11-1006G14.1 | - | -1.11 | 0.02361 |
| COL9A2 | collagen type IX alpha 2 chain | -1.11 | 0.00313 |
| UNC5D | unc-5 netrin receptor D | -1.12 | 0.01434 |
| RP11-136C24.1 | - | -1.12 | 0.04840 |
| THBS3 | thrombospondin 3 | -1.12 | 0.00316 |
| FAM179A | - | -1.13 | 0.04941 |
| HSPA12B | heat shock protein family A (Hsp70) member 12B | -1.14 | 0.00042 |
| MAN1B1-AS1 | - | -1.14 | 0.00985 |
| MICAL2 | microtubule associated monooxygenase, calponin and LIM domain containing 2 | -1.14 | 0.00124 |
| SYN3 | synapsin III | -1.15 | 0.02913 |
| SCN4B | sodium voltage-gated channel beta subunit 4 | -1.15 | 0.00105 |
| HCN2 | hyperpolarization activated cyclic nucleotide gated potassium channel 2 | -1.19 | 0.03067 |
| MT-ND5 | mitochondrially encoded NADH:ubiquinone oxidoreductase core subunit 5 | -1.21 | 0.04162 |
| RP11-328C8.5 | - | -1.21 | 0.03606 |
| RP11-676J12.8 | - | -1.21 | 0.04833 |
| XKR5 | XK related 5 | -1.21 | 0.00741 |
| NANOS1 | nanos C2HC-type zinc finger 1 | -1.21 | 0.02524 |
| UNC13A | unc-13 homolog A | -1.23 | 0.02445 |
| LRRTM1 | leucine rich repeat transmembrane neuronal 1 | -1.23 | 0.02102 |
| LOXL2 | lysyl oxidase like 2 | -1.23 | 0.00079 |
| TBX4 | T-box 4 | -1.23 | 0.02361 |
| GABRD | gamma-aminobutyric acid type A receptor delta subunit | -1.24 | 0.02008 |
| TBX1 | T-box 1 | -1.24 | 0.02076 |
| TCEAL7 | transcription elongation factor A like 7 | -1.24 | 0.01372 |
| STXBP6 | syntaxin binding protein 6 | -1.25 | 0.00627 |
| ASPN | asporin | -1.25 | 0.00353 |
| CDH7 | cadherin 7 | -1.26 | 0.02610 |
| HOXD9 | homeobox D9 | -1.27 | 0.00241 |
| GABRG3 | gamma-aminobutyric acid type A receptor gamma3 subunit | -1.27 | 0.02876 |
| ITPKA | inositol-trisphosphate 3-kinase A | -1.30 | 0.00614 |
| RP11-283G6.3 | - | -1.30 | 0.02302 |
| ST8SIA5 | ST8 alpha-N-acetyl-neuraminide alpha-2,8-sialyltransferase 5 | -1.31 | 0.02215 |
| C1QTNF2 | C1q and tumor necrosis factor related protein 2 | -1.32 | 0.00079 |
| TRIM71 | tripartite motif containing 71 | -1.33 | 0.00042 |
| RASA4 | RAS p21 protein activator 4 | -1.33 | 0.00316 |
| PALM3 | paralemmin 3 | -1.34 | 0.03257 |
| AGBL1 | ATP/GTP binding protein like 1 | -1.34 | 0.02100 |
| AXDND1 | axonemal dynein light chain domain containing 1 | -1.35 | 0.01178 |
| PSD2 | pleckstrin and Sec7 domain containing 2 | -1.36 | 0.01428 |
| SEMA3A | semaphorin 3A | -1.36 | 0.00328 |
| TRIM36 | tripartite motif containing 36 | -1.36 | 0.02400 |
| FAXC | failed axon connections homolog | -1.37 | 0.00766 |
| IGFN1 | immunoglobulin-like and fibronectin type III domain containing 1 | -1.38 | 0.01120 |
| ANGPTL2 | angiopoietin like 2 | -1.38 | 0.00710 |
| XPNPEP2 | X-prolyl aminopeptidase 2 | -1.38 | 0.00395 |
| NPFFR1 | neuropeptide FF receptor 1 | -1.41 | 0.02835 |
| SNHG23 | - | -1.41 | 0.01428 |
| TPPP3 | tubulin polymerization promoting protein family member 3 | -1.41 | 0.01468 |
| RP11-554A11.4 | - | -1.43 | 0.00492 |
| RAB3B | RAB3B, member RAS oncogene family | -1.43 | 0.01438 |
| CSMD2 | CUB and Sushi multiple domains 2 | -1.44 | 0.02278 |
| TSSC2 | - | -1.44 | 0.01485 |
| LINC01122 | - | -1.44 | 0.02489 |
| RP11-595O22.1 | - | -1.44 | 0.00254 |
| SLC12A1 | solute carrier family 12 member 1 | -1.45 | 0.00106 |
| MEG8 | - | -1.45 | 0.00985 |
| NHLH2 | nescient helix-loop-helix 2 | -1.46 | 0.03244 |
| DPF1 | double PHD fingers 1 | -1.49 | 0.00219 |
| PDLIM3 | PDZ and LIM domain 3 | -1.51 | 0.00152 |
| CACNG4 | calcium voltage-gated channel auxiliary subunit gamma 4 | -1.52 | 0.01372 |
| RP3-323A16.1 | - | -1.53 | 0.01376 |
| DNAH6 | dynein axonemal heavy chain 6 | -1.53 | 0.01009 |
| ADAMTS3 | ADAM metallopeptidase with thrombospondin type 1 motif 3 | -1.56 | 0.02210 |
| IGDCC3 | immunoglobulin superfamily DCC subclass member 3 | -1.56 | 0.00078 |
| NUP210L | nucleoporin 210 like | -1.57 | 0.04941 |
| INA | internexin neuronal intermediate filament protein alpha | -1.59 | 0.01502 |
| VWDE | von Willebrand factor D and EGF domains | -1.60 | 0.01914 |
| IGSF10 | immunoglobulin superfamily member 10 | -1.60 | 0.01631 |
| PIANP | PILR alpha associated neural protein | -1.63 | 0.00628 |
| RP11-142J21.2 | - | -1.64 | 0.02876 |
| LRP2 | LDL receptor related protein 2 | -1.65 | 0.01376 |
| PAEP | progestagen associated endometrial protein | -1.67 | 0.00152 |
| CSMD1 | CUB and Sushi multiple domains 1 | -1.70 | 0.04287 |
| ELOVL2 | ELOVL fatty acid elongase 2 | -1.70 | 0.03271 |
| MDGA2 | MAM domain containing glycosylphosphatidylinositol anchor 2 | -1.76 | 0.00092 |
| NTF3 | neurotrophin 3 | -1.77 | 0.00199 |
| TNN | tenascin N | -1.81 | 0.00002 |
| SSC5D | scavenger receptor cysteine rich family member with 5 domains | -1.81 | 0.00119 |
| TFAP2B | transcription factor AP-2 beta | -1.89 | 0.00141 |
| JPH4 | junctophilin 4 | -1.90 | 0.00001 |
| NFATC2 | nuclear factor of activated T-cells 2 | -2.01 | 0.00008 |
| FAIM2 | Fas apoptotic inhibitory molecule 2 | -2.02 | 0.00816 |
| POSTN | periostin | -2.08 | 0.00003 |
| FREM2 | FRAS1 related extracellular matrix protein 2 | -2.16 | 0.00009 |
| GAD1 | glutamate decarboxylase 1 | -2.19 | 0.00019 |
| PCDH11X | protocadherin 11 X-linked | -2.21 | 0.00020 |
| KCNJ6 | potassium voltage-gated channel subfamily J member 6 | -2.29 | 0.00004 |
| KCNA1 | potassium voltage-gated channel subfamily A member 1 | -2.37 | 0.00247 |

**Supplementary Table 2.** Pathways predicted to be activated (z-score ≥1.7) or inhibited (z-score ≤-1.7) in infraocclusion using Ingenuity Pathway Analysis. A z-score of ≥ 2 or ≤ -2 indicates a prediction of high confidence; an empirical cut-off of 1.7 was applied, as a cut-off of 2 excluded relevant related functions, and a lower cut-off included more non-specific functions with decreasing confidence.

| Pathway | Figure illustration | p-value | Predicted Activation State | Activation z-score | Number of DE genes | DE genes |
| --- | --- | --- | --- | --- | --- | --- |
| Inflammatory response | Figure 3 | 0.005 | Increased (high confidence) | 2.78 | 16 | BPI, C3, CCL28, CXADR, CXCL1, F11R, F2RL1, IL18, KLKB1, ODAM, PLAT, RORA, S100A12, S100A8, S100A9, VNN1 |
| Cellular homeostasis | Not illustrated (non-specific) | 0.006 | Increased (high confidence) | 2.75 | 33 | ABHD5, AQP1, AQP3, ATG9B, C3, CCL28, CLCA2, CXCL1, F11R, F2RL1, FCGR3A/FCGR3B, GPR182, HCN2, IL18, JPH4, KCNA1, LAMA3, LRRK2, MLXIPL, MYADM, MYCN, PDK4, PYGL, RHCG, S100A8, S100A9, SPINK5, STOX1, TGFA, TIMP2, TNFSF10, TP63, XDH |
| Movement of blood cells | Figure 3 | 0.005 | Increased (high confidence) | 2.55 | 17 | ANGPTL2, C3, CCL28, CXADR, CXCL1, F11R, F2RL1, FCGR3A/FCGR3B, IL18, MYADM, PAEP, S100A12, S100A8, S100A9, SEMA3A, SLPI, TIMP2 |
| Interaction of blood cells | Figure 3 | 0.001 | Increased (high confidence) | 2.49 | 15 | ADRB2, BPI, C3, CCL28, CD80, CDH1, F11R, FCGR3A/FCGR3B, LTF, MYADM, S100A8, S100A9, SEMA3A, ST6GALNAC2, TLR5 |
| Binding of blood cells | Figure 3 (subset of interaction of blood cells) | 0.002 | Increased (high confidence) | 2.33 | 14 | ADRB2, BPI, C3, CCL28, CDH1, F11R, FCGR3A/FCGR3B, LTF, MYADM, S100A8, S100A9, SEMA3A, ST6GALNAC2, TLR5 |
| Growth of epithelial tissue | Figure 4 | 0.000 | Increased (high confidence) | 2.26 | 23 | BMP5, BPI, C3, CDH3, CEACAM1, CXCL1, F11R, F2RL1, FABP4, FERMT1, IL1RN, LAMA3, LCN2, LMNA, LOXL2, MMP12, RGS4, TGFA, TGM1, TIMP2, TNFSF10, TP63, XDH |
| Endothelial cell apoptosis | Figure 3 | 0.002 | Increased (high confidence) | 2.22 | 5 | IL18, MMP2, S100A8, S100A9, TNFSF10 |
| Adhesion of blood cells | Figure 3 (subset of binding of blood cells) | 0.004 | Increased (high confidence) | 2.13 | 12 | ADRB2, C3, CCL28, CDH1, F11R, FCGR3A/FCGR3B, LTF, MYADM, S100A8, S100A9, ST6GALNAC2, TLR5 |
| Aggregation of cells | Not illustrated (non-specific) | 0.005 | Increased (high confidence) | 2.13 | 10 | ALOX12, ANGPTL2, CDH1, CDH3, DSC2, DSG2, PLAT, PLPP3, SEMA3A, TIMP2 |
| Epithelial cell differentiation | Figure 4 | 0.000 | Increased (moderate confidence) | 1.98 | 15 | AQP3, CDH1, CERS3, CSTA, DSP, EVPL, SCEL, SFN, SH3PXD2A, SPINK5, SPRR1B, ST14, TGFA, TGM1, TP63 |
| Keratinocyte differentiation | Figure 4 (subset of epithelial cell differentiation) | 0.000 | Increased (moderate confidence) | 1.98 | 14 | AQP3, CDH1, CERS3, CSTA, DSP, EVPL, SCEL, SFN, SH3PXD2A, SPRR1B, ST14, TGFA, TGM1, TP63 |
| Immune response of neutrophils | Figure 3 | 0.005 | Increased (moderate confidence) | 1.98 | 4 | C3, CXCL1, FCGR3A/FCGR3B, IL18 |
| Formation of skin | Figure 4 | 0.000 | Increased (moderate confidence) | 1.96 | 26 | AQP3, CDH1, CDH3, CERS3, COL17A1, COL5A1, CSTA, DSP, EVPL, FABP5, KRT14, KRT15, KRT16, KRT5, LAMA3, LAMB3, LAMC2, SCEL, SFN, SH3PXD2A, SPINK5, SPRR1B, ST14, TGFA, TGM1, TP63 |
| Cell movement | Not illustrated (non-specific) | 0.000 | Increased (moderate confidence) | 1.91 | 85 | ADRB2, ALOX12, ANGPTL2, ANGPTL4, ANXA3, C3, CCL28, CDCP1, CDH1, CDH3, CEACAM1, CHL1, CHRD, CLCA2, CLDN7, COL17A1, CRY1, CXADR, CXCL1, DSG3, DSP, EHF, EPHA1, ESRP1, F11R, F2RL1, FAM110C, FCGR3A/FCGR3B, FERMT1, FGD4, FLNC, HOOK1, HTR7, IL18, ITGB6, KIF26B, KLF5, KRT16, KRT19, LAMA3, LAMB3, LAMC2, LCN2, LMNA, LOXL2, MME, MMP12, MMP19, MMP2, MYADM, NFATC2, NTF3, OSGIN1, PAEP, PARD6B, PITX2, PLAT, PLPP3, POSTN, RGS4, S100A12, S100A2, S100A8, S100A9, S100B, S100P, SEMA3A, SERPINB5, SFN, SH3PXD2A, SLC9A3R1, SLPI, SPINT1, ST6GALNAC2, STAB2, TACSTD2, TGFA, TIMP2, TNFSF10, TNN, TNS4, TP63, TPD52L1, VAV3, XDH |
| Migration of cells | Not illustrated (non-specific) | 0.000 | Increased (moderate confidence) | 1.83 | 80 | ADRB2, ALOX12, ANGPTL4, ANXA3, C3, CCL28, CDCP1, CDH1, CDH3, CEACAM1, CHL1, CHRD, CLCA2, CLDN7, COL17A1, CRY1, CXADR, CXCL1, DSG3, DSP, EHF, EPHA1, ESRP1, F11R, F2RL1, FAM110C, FCGR3A/FCGR3B, FERMT1, FLNC, HTR7, IL18, ITGB6, KIF26B, KLF5, KRT16, KRT19, LAMA3, LAMB3, LAMC2, LCN2, LMNA, LOXL2, MME, MMP12, MMP19, MMP2, MYADM, NFATC2, NTF3, OSGIN1, PAEP, PARD6B, PITX2, PLAT, PLPP3, POSTN, RGS4, S100A12, S100A2, S100A8, S100A9, S100B, S100P, SEMA3A, SERPINB5, SFN, SLC9A3R1, SLPI, SPINT1, STAB2, TACSTD2, TGFA, TIMP2, TNFSF10, TNN, TNS4, TP63, TPD52L1, VAV3, XDH |
| Epithelial cell proliferation | Figure 4 | 0.003 | Increased (moderate confidence) | 1.73 | 10 | BMP5, CDH3, FERMT1, LAMA3, LMNA, MMP12, RGS4, TGFA, TGM1, TP63 |
| Cell-cell contact | Not illustrated (non-specific) | 0.000 | Increased (moderate confidence) | 1.72 | 20 | CDH1, CDH3, CLDN1, CRNN, CSTA, CXADR, DSG3, DSP, F11R, GJB2, GJC1, LRRK2, PARD6B, PTGER3, SH3PXD2A, SPINT2, TP63, UNC5D, VNN1, XDH |
| Keratinocyte interaction | Figure 4 | 0.000 | Increased (moderate confidence) | 1.72 | 6 | CDH1, CDH3, DSG3, FERMT1, KRT16, SH3PXD2A |
| Epithelial cell movement | Figure 4 | 0.000 | Decreased (moderate confidence) | -1.94 | 9 | ADRB2, CDH1, COL17A1, FERMT1, KRT16, LAMA3, RGS4, SH3PXD2A, TP63 |
| Keratinocyte cell movement | Figure 4 (subset of cell movement of epithelial cells) | 0.000 | Decreased (high confidence) | -2.21 | 7 | ADRB2, COL17A1, FERMT1, KRT16, LAMA3, SH3PXD2A, TP63 |
